# Identification of host interactions for phenotypic antimalarial hits

**DOI:** 10.1186/1758-2946-6-S1-O12

**Published:** 2014-03-11

**Authors:** Andreas Spitzmüller, Jordi Mestres

**Affiliations:** 1 Chemotargets SL and Systems Pharmacology Group, Research Programme on Biomedical Informatics (GRIB), IMIM Hospital del Mar Research Institute and Universitat Pompeu Fabra, Parc de Recerca Biomèdica, Doctor Aiguader 88, 08003 Barcelona, Catalonia, Spain

## 

Malaria is one of the most epidemic infectious diseases in the world affecting millions of patients and causing more than 500,000 deaths each year. Although there are several established antimalarials in clinical use, there is an urgent need for new drugs due to rapid resistance development. In recent years, more than 20,000 hits phenotypically active against *P. falciparum*, one of the major malaria causing agents, were disclosed from three independent HTS campaigns [1-3]. In order to make these hit libraries accessible to as many biological laboratories as possible, the MMV compiled and distributed the Open Access Malaria Box, a set of 400 chemically diverse active compounds [4]. One important task is now to elucidate the mode of action of those compounds. However, beside the parasite targets it is also necessary to identify potential host interactions in order to anticipate the risk of undesired side effects of those chemotypes at the earliest possible stage of development.

To this end, we applied a ligand-based virtual target profiling approach to predict possible interactions with human targets [5]. Amongst others, kinases and GPCRs were identified as the most important target classes. Subsequently, several hundred predicted interactions were selected for prospective experimental testing. Results showed that a substantial part of the Malaria Box exhibits the potential of interacting with human GPCRs. To this extent, this was unexpected beforehand since the pathogenic agent does not contain any GPCRs. In this respect, particular attention was given to 5-HT2B receptor agonism, an effect associated to cardiac valvulopathy [6].
